# Metastasizing Bronchopulmonary Leiomyosarcoma

**DOI:** 10.1177/2324709615584000

**Published:** 2015-04-22

**Authors:** Speros Livieratos, Eddie Fatakhov, Ali Ammar, Thomas Dillard, Bruce Davis

**Affiliations:** 1 Georgia Regents University, Augusta, GA, USA; 2 UCLA-Kern Medical Center, Bakersfield, CA, USA

**Keywords:** metastasizing, bronchopulmonary, leiomyosarcoma, cancer, uterine fibroids, pleural fluid

## Abstract

An otherwise healthy 55-year-old female, nonsmoker, was seen in pulmonary consultation for progressively worsening shortness of breath. She had undergone a complete hysterectomy 7 years prior for bleeding leiomyomas. On presentation, her initial chest X-ray showed a large right-sided pleural effusion with multiple pulmonary nodules. Two thoracenteses failed to reveal any cytologic abnormalities. Bronchoscopy revealed smooth, round, endobronchial lesions. Histologic examination showed features consistent with leiomyosarcoma. We present a rare case of a patient that initially had possible leiomyomas of the uterus surgically removed and years later presented with bronchopulmonary leiomyosarcoma.

## Case Report

**Figure 1.**
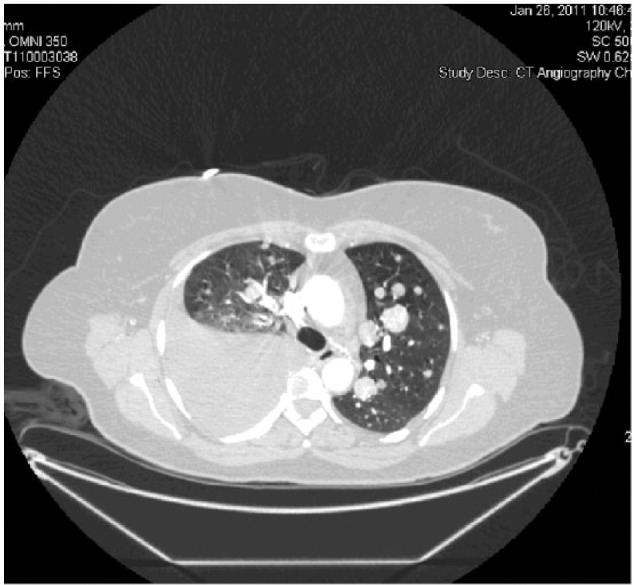
Computed tomography scan of the chest, abdomen, and pelvis revealed too numerous to count bilateral pulmonary nodules with a large pleural effusion, and near complete collapse of the right middle and right lower lobes.

**Figure 2.**
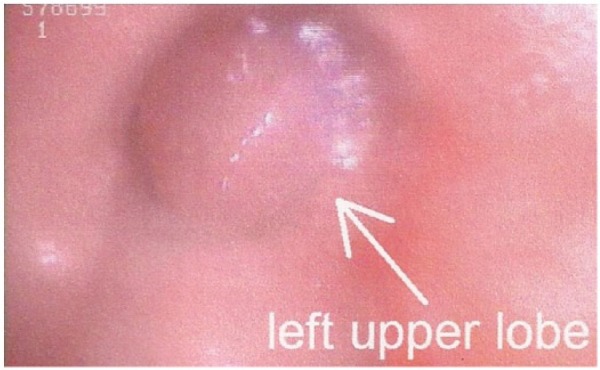
Friable airway lesion seen on bronchoscopy.

**Figure 3.**
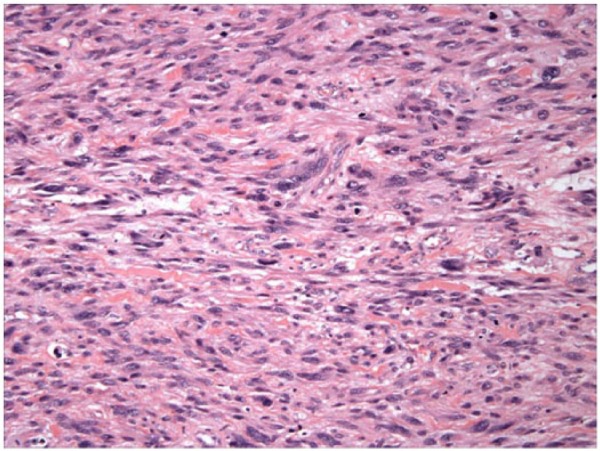
Pathology results of the video-assisted thoracoscopic surgery showed actin and desmin confirming a low-grade leiomyosarcoma.

**Figure 4.**
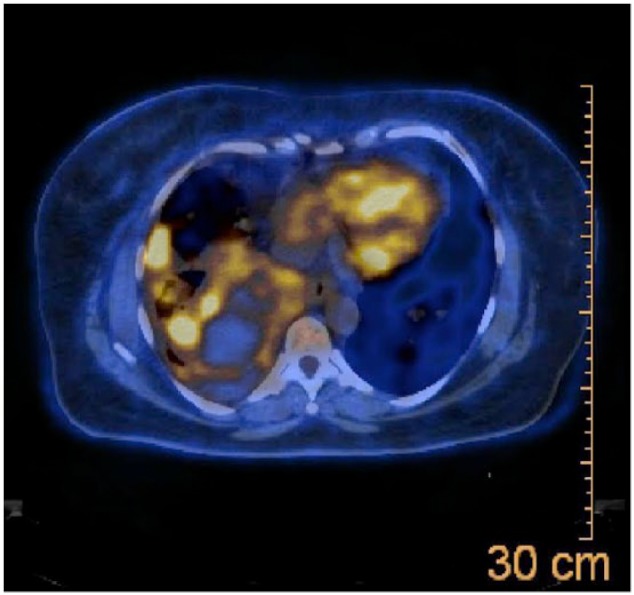
Positron emission tomography–computed tomography scan of chest showing multiple pulmonary nodules.

We present a case of a 55-year-old female that presented with a 3-week history of shortness of breath, cough, and right-sided pleuritic chest pain. The patient was a nonsmoker and had a significant past medical history of complete hysterectomy 7 years prior for bleeding uterine fibroids. Pathology at that time had shown the fibroids to be leiomyomas. On current presentation, her chest X-ray showed multiple pulmonary nodules with a large right-sided pleural effusion. Subsequently, a computed tomography scan of the chest, abdomen, and pelvis revealed numerous bilateral pulmonary nodules with a large pleural effusion, and near complete collapse of the right middle and right lower lobes (Figure 1). The sizes of the nodules was not documented but had borders that were circular and distinct. There were no abnormal lesions seen under the diaphragm. Two thoracenteses revealed bloody, exudative, pleural fluid but cytology failed to reveal any evidence of malignancy. Bronchoscopy of the right lung revealed extrinsic compression of the airways in the right middle and right lower lobe with no intrabronchial lesions. However, there was a large nonendobronchial mass on the right side causing atelectasis and collapse. Being the focus of the bronchoscopy, the left lung revealed 2 endobronchial lesions: one in the apicoposterior segment of the left upper lobe and one within the basilar segments of the left lower lobe. The lesions were smooth, round, and freely mobile with the patient’s inspiratory and expiratory effort. Each was anchored to the inside of the airways by thin stalks (Figure 2). Biopsy of one of these lesions showed the lesions to be very friable. Histopathology revealed proliferating spindle cells that were highlighted with smooth muscle actin and desmin (Figure 3). With the pleural effusion returning, the patient subsequently underwent a video-assisted thoracoscopic surgery (VATS) procedure to obtain more tissue for further histologic investigation and symptomatic relief. Pathology results of the VATS revealed a low-grade leiomyosarcoma. The patient was referred to Hematology/Oncology, and a positron emission tomography and computed tomography scan (PET-CT) was completed. The PET-CT scan showed bilateral, innumerable hypermetabolic masses in both lungs (Figure 4). There was also bilateral mediastinal and hilar lymphadenopathy, which had no significant uptake. In addition, there was a multiloculated, mildly active, pleural effusion. The patient was felt to be a poor surgical candidate due to the vast number of pulmonary tumors present. She was started on a course of chemotherapy consisting of docetaxel (taxotere) and gemcitabine (gemzar). As of June 31, 2011, the patient has been tolerating chemotherapy with minimal side effects and was scheduled for a follow-up PET-CT in July 2011.

## Discussion

Bronchopulmonary leiomyosarcoma is a rare entity that can occur in males and females of varying age groups.^1^ Both primary tumors and metastatic disease have been reported.^1,2^ If present in the uterus, these lesions can cause excessive uterine bleeding and surgical intervention is needed. Bronchopulmonary leiomyosarcoma can have a very unpredictable clinical course as a primary tumor in the lung, or as a metastatic focus to the lung from another source. Even when surgically removed, metastatic seeding can occur and remain dormant for many years before declaring itself. In even rarer cases, leiomyosarcoma can coexist with benign fibroid tumors called leiomyomas.^3^ These benign fibroid tumors occur in reproductive-aged females and can cause excessive uterine bleeding sometimes requiring surgical intervention. Metastatic seeding of these tumors is also rare but has been reported in case reports.^3^ There is some suggestion in the literature that leiomyomas may be a type of low-grade leiomyosarcoma but this point is of great debate.^4^

Leiomyosarcoma is a rare entity. Out of all the pulmonary tumors, pulmonary sarcomas comprise 0.5% of lung neoplasms. Leiomyosarcomas are the most common sarcomas of the lung.^5^ They are characterized by prominent cellular atypia, greater than 10 mitoses per 10 high power fields, and areas of tumor cell necrosis. These 3 characteristics have been deemed the Stanford criteria. The presence of 2 or more of these criteria represents a greater than 10% risk of metastatic spread.^6^

Pulmonary leiomyosarcoma case reports are rare in the literature. For every 3000 cases of pulmonary carcinomas, there is 1 case of pulmonary leiomyosarcoma.^2^ In a case report from 1957, twelve documented cases were quoted.^1^ In a 2002 case report, 92 cases worldwide were quoted.^2^ These tumors are initially detected on radiography and commonly arise in the left lower lobe,^7^ metastasize very late in their course, and spread hematogenously. When leiomyosarcoma metastasizes to the lung, the tumor is usually centrally located and can be often seen in the major bronchi. Grossly, this lesion has been described as a finger-like, fungating projection with little invasion of local lung parenchyma. The tumor itself is white to red in appearance, very friable, and bleeds easily. Depending on the tumor size, it can fill part or all of the bronchial opening.^1^ According to Naik et al,^5^ these tumors, unlike epithelial tumors, do not exhibit any exfoliating tendencies and therefore bronchoscopy and washings or even pleural fluid collection may not yield much diagnostic information.

A similar entity, benign metastasizing leiomyoma, was first described in 1939.^4^ By 2001, there were about 75 documented cases.^3^ Leiomyosarcomas and leiomyomas are separate entities. Both processes can coexist in the same patient but they exhibit different pathologic and cytogenetic properties.^6^ In benign metastasizing leiomyoma, pulmonary nodules can be seen 3 months to 20 years after hysterectomy due to its slow and indolent course.^3^ Histologically, leiomyomas consist of well-differentiated stroma, with a benign appearance of smooth muscle morphology. Some authors have suggested that these lesions represent metastatic low-grade leiomyosarcoma but that point is still of great debate in the literature.^4^

For this case, it was determined by the tumor board that the lung involvement was part of a metastatic process. This assessment is based on the clinical history, the patient’s previous fibroid history, and the comparison of the pathology slides from 7 years ago and present time. The reason this case was reported is the fact that this particular malignancy can lie dormant, even when surgically removed.

In conclusion, bronchopulmonary leiomyosarcoma is very rare, very unpredictable and can clinically declare itself within a few months to many years after surgical resection. In rare cases, it can be present in patients that have been diagnosed with benign leiomyomas of the uterus.

## Follow-up

The patient’s previous histopathology slides were reviewed and compared with the more recent slides, and although it is not official, there is a possibility that the initial lesion 7 years ago was a low-grade leiomyosarcoma. In review of the original slides 7 years prior, it was decided that the original diagnosis of uterine fibroids was actually leiomyosarcoma. However, there the original tissue samples to confirm this were not available but tissue from the present-day lung was compared with the original slides. The pathologist, oncologist, and rest of the tumor board concluded that the primary was likely the uterine fibroids from 7 years ago due to the similarities seen between them.

The patient had a follow-up PET-CT scan that showed stable-sized pulmonary nodules with decreased metabolic activity. The large, dominant mass showed increased necrosis with decreased metabolic activity. There was a smaller, residual pleural effusion with no metabolic uptake and nonspecific mediastinal and retroperitoneal lymphadenopathy also with no metabolic uptake. As of August 2011, the patient continues to have weekly chemotherapy and is slated to have serial PET-CT scans. However, patient did not respond to chemotherapy and subsequently passed away in 2012.
